# Iliac branch endoprosthesis for endovascular treatment of complex aorto‐iliac aneurysms – from device design to practical experience: how to translate physiology considerations into clinical applications

**DOI:** 10.1113/EP091801

**Published:** 2024-11-29

**Authors:** Apostolos G. Pitoulias, Mario D΄Oria, Konstantinos P. Donas, Matti Jubouri, Damian M. Bailey, Ian M. Williams, Mohamad Bashir

**Affiliations:** ^1^ Rhein Main Vascular Center, Department of Vascular and Endovascular Surgery, Asklepios Clinics Langen Paulinen Wiesbaden Seligenstadt Germany; ^2^ Division of Vascular and Endovascular Surgery, Department of Clinical Surgical and Health Sciences University of Trieste Trieste Italy; ^3^ Hull York Medical School University of York York UK; ^4^ Neurovascular Research Laboratory, Faculty of Life Sciences and Education University of South Wales Pontypridd UK; ^5^ Department of Vascular Surgery University Hospital of Wales Cardiff UK; ^6^ Vascular & Endovascular Surgery, Velindre University NHS Trust Health & Education Improvement Wales (HEIW) Cardiff UK

**Keywords:** aortic aneurysm, endovascular repair, hypogastric artery, iliac aneurysm, iliac branch, pelvic circulation, stent‐graft

## Abstract

This article provides a narrative review of the current literature and our expert opinion concerning the iliac branch endoprosthesis (IBE) and its use in the treatment of complex abdominal aortic aneurysm (AAA) cases with concomitant aneurysmal involvement of the common iliac artery (CIA) and/or the internal iliac artery (IIA). Up to 25% of those with an AAA may present with extension of the aneurysmal disease into the iliac vessels. This anatomy may complicate the standard endovascular aortic repair (EVAR) procedure, as the available length of distal landing zones is altered. The optimum treatment requires both the adequate sealing of the distal landing zone as well as the preservation of the pelvic circulation through the IIA. Extensive preoperative assessment of the anatomy, as well as an accurate deployment following all procedural steps, enables endovascular treatment of complex aorto‐iliac aneurysms safe with excellent midterm clinical outcomes. The current literature shows that the utilization of the IBE offers a durable treatment of these complicated cases with results equal to those of the open repair, without the associated morbidity. Preservation of the pelvic circulation is recommended to prevent pelvic ischaemic symptoms and can also be carried out on both sides provided certain anatomical requirements are met.

## Abstract (highlights)

- **What is the topic of this review?**
  How iliac branch devices (IBD) can extend technical options for endovascular treatment of aortoiliac aneurysms and whether lifelong dual antiplatelet therapy (DAPT) is routinely required for increasing patency rates after implantation of IBD.
- **What advances does it highlight?**
  IBD are versatile devices that can safely be implanted also outside standard instructions‐for‐use such as in cases with hypogastric aneurysms. While lifelong DAPT may not be routinely advised following IBD implantation, its use is usually recommended in the first 3–6 months but may be extended in selected circumstances.

## 1 INTRODUCTION

An abdominal aortic aneurysm (AAA) can be defined as a dilatation of the abdominal aorta with a diameter of more than 3 cm (Owens et al., 2019). The prevalence of the disease ranges from 2% to 18.5% for men and from 0.1% to 4.2% for women (Al‐Balah et al., 2020). Since the first endovascular aneurysm repair (EVAR) in 1990 (Parodi, 1995), EVAR has become established as a routine procedure to treat AAA. After 30 years of innovations, medical technology has enabled the manufacture of devices to treat complex AAA with extension of disease both suprarenally and into the iliac arteries (Wanhainen et al., 2024).

There are many anatomical requirements specific for each device to allow them to remain within the manufacturers' instructions‐for‐use (IFU). For a durable seal, the length and diameter of the proximal and distal landing zones should be of a suitable length and width. While the anatomical requirements may be slightly different between different manufacturers (and a specific description of the IFU for each device lies beyond the purpose of this work), most require at least 10 mm (or 15 mm in some cases) of sealing length in a healthy area that is not conical or enlarged and free from significant atherothrombotic burden. It is becoming increasingly apparent the deployment of an endograft outside its intended IFU can lead to higher rates of endoleaks and reinterventions (D'Oria & Galeazzi, 2022).

Up to 25% of those with an AAA may have an associated common iliac artery (CIA) aneurysm which can involve the internal iliac artery (IIA) (Parlani et al., 2002). This situation may reduce the available distal landing zone and limit chances of a successful EVAR. Open repair has always been the solution for these AAA with complex anatomies but can be associated with increased periprocedural complications (Patel et al., 2016). For the endovascular treatment of complicated AAA, three dedicated devices have been developed. To date, the Gore Excluder Iliac Branch Endoprosthesis (IBE) (W. L. Gore & Associates, Inc., Newark, DE, USA) is the only US Food and Drug Administration (FDA)‐approved, off‐the‐shelf device which manages to preserve unobstructed blood flow to the IIA and offer an adequate sealing at the distal landing zone.

Preservation of pelvic blood flow through the IIA is an important aspect of a successful EVAR procedure. Historically, coverage of the IIA ostium with embolization of the hypogastric trunk to limit the risk of endoleak was common practice. However, the consequences of this can vary from buttock claudication and erectile dysfunction to intestinal and spinal cord ischaemia (SCI) (Bekdache et al., 2015; Bosanquet et al., 2017; Lin et al., 2009). A compromise with adequate results is the unilateral coverage of IIA during EVAR (Mazzaccaro et al., 2019). De Athayde Soares et al. (2023) showed in their study the crucial role of the number of patent IIA at the end of an EVAR for the preservation of pelvic blood flow. This showed 94% undergoing bilateral IIA coverage had evidence of bowel ischaemia compared to only 6% of those with unilateral coverage. No cases of bowel ischaemia were documented in the group without IIA coverage (de Athayde Soares et al., 2023). The most significant complication of extensive fenestrated‐branched EVAR for treatment of thoracoabdominal aortic disease is SCI. There is also a correlation with coverage of IIA as the pelvic circulation is known to be a critical source of collateral flow to the spinal cord (Eagleton et al., 2014). From a physiological standpoint, it is absolutely mandatory to preserve the pelvic circulation to the best extent possible when extensive aortic coverage is being planned or is likely to be required in the future.

## 2 DEVICE DESCRIPTION AND PROCEDURAL STEPS

The development of the IBE endoprothesis provides a safe and effective solution for the preservation of IIA while simultaneously providing adequate sealing of the distal landing zone. However, strict adherence to the IFU tends to reduce its widespread usage. Multicentre studies have shown less than 65% of patients with involvement of IIA meet the anatomical requirements for the treatment with iliac branch devices (IBDs). However, a more liberal approach with the IFU may enable these devices to be deployed in 96% (Gouveia et al., 2022; Pearce et al., 2015).

The Gore Excluder IBE is a bifurcated stent‐graft, made from expanded polytetrafluoroethylene (ePTFE) fabric mounted on a nitinol stent frame. It consists of two components, the iliac branch component (IBC) and the internal iliac component (IIC). The IBC is a bifurcated device that fits within a 16Fr sheath, with a leg for the external iliac artery and a portal for the IIA. The IBC component has a proximal diameter of 23 mm, and an overall length of 100 mm, and the available distal diameters are 10, 12 and 14.5 mm, respectively. The IIC component has a proximal diameter of 16 mm, a length of 70 mm, and the same distal diameter range as the IBC component, (i.e., 10, 12 and 14.5 mm).

**FIGURE 1:**
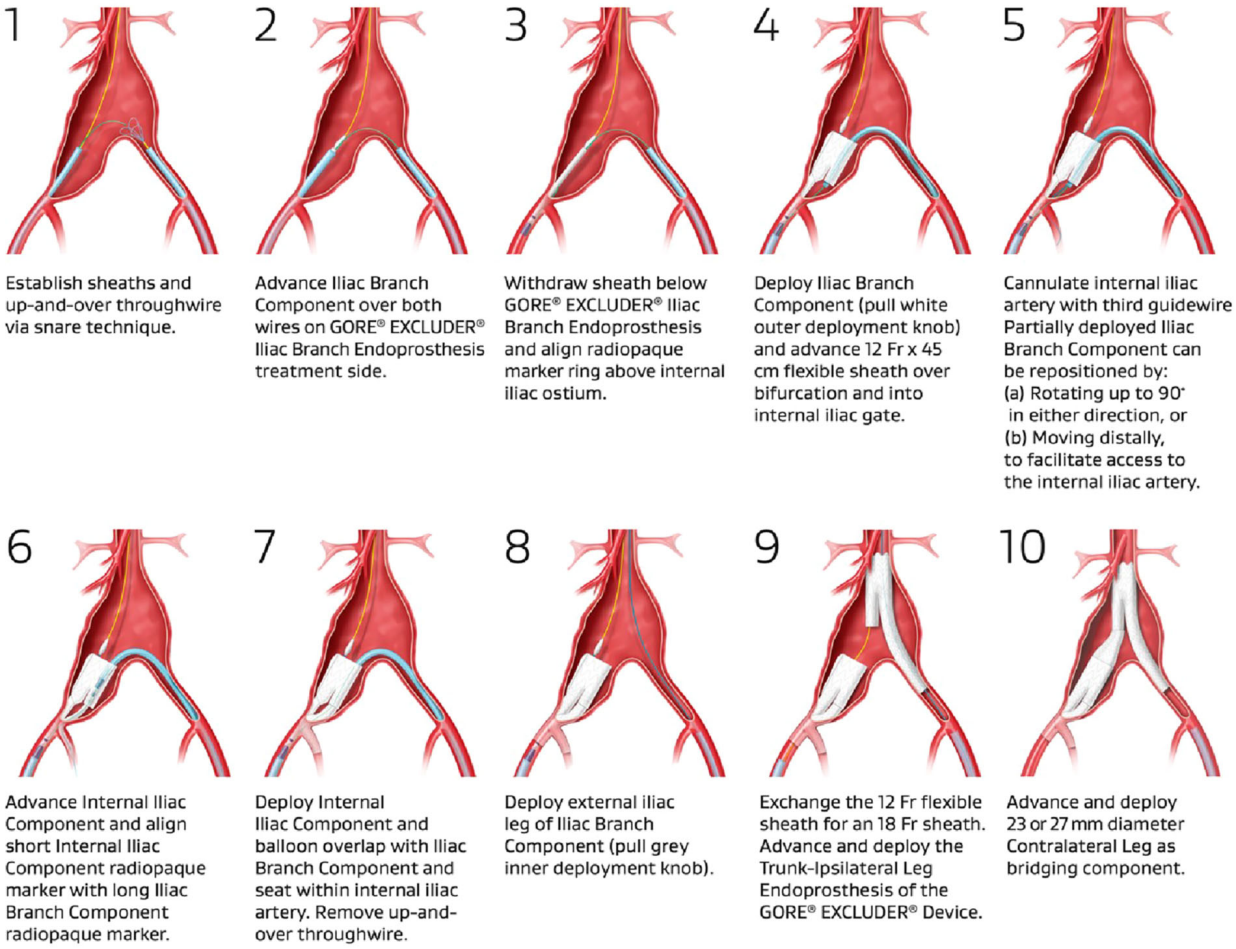
Deployment of IBE device (Gore Excluder Iliac Branch Endoprothesis).

According to IFU, for the implantation of the IBC, a minimum CIA diameter of 17 mm at the proximal landing zone is required. For the distal landing zone at the external iliac artery, a length of 30 mm and a diameter of 6.5–13.5 mm is required. For the IIC a length of 30 mm and a diameter of 6.5–13.5 mm at the distal landing zone is required. Once the IBC has been deployed, catheterization of the IIA is performed from a contralateral approach and the IIC is implanted. Following the release of the main aortic stent‐graft, the whole operation is concluded with the advancement and deployment of a 23 or 27 mm bridging iliac limb from the ipsilateral side. A minimum longitudinal distance between the gate of the main aortic stent‐graft and the proximal part of the IBC is required to allow for effective implantation, and this distance is even for bilateral applications. This remains a notable limitation to bilateral implantation in some cases, although off‐label techniques have been reported to address the issue and expand the device applicability, such as the use of shorter bridging limbs from different manufacturers. Figure 1 shows the main intraprocedural steps for the implantation of the device.

## 3 CLINICAL EXPERIENCE

Open surgery, with its technical challenges and increased peri‐ and postoperative complications (Cochennec et al., 2010), was the only solution for those without adequate distal landing zone. This enabled effective preservation of pelvic blood flow and concomitant exclusion of the aneurysm. Considering the endovascular approach, EVAR with adjunctive exclusion of the IIA using coils or plugs (and provisional extension of the distal landing zone within the external iliac artery) was suggested as an alternative to the use of flared iliac limbs (also known as the bell‐bottom technique) to avoid the risk for late type 1B endoleaks. Bosanquet et al. (2017) in their meta‐analysis showed that one in three patients undergoing EVAR with exclusion of the IIA may develop buttock claudication and one in ten male patients may suffer from new‐onset erectile dysfunction. Since the development of the IBDs, the spectrum of endovascular treatment for AAA has been extended and now includes on‐label totally endovascular incorporation of the pelvic arteries in the repair in most cases. In effect, a recent meta‐analysis by Giosdekos et al. (2020) demonstrated that the use of IBDs is highly safe, with excellent outcomes at least over midterm follow‐up.

A review of the current literature reveals a plethora of evidence supporting the effectiveness and safety of the Gore Excluder IBE for treatment of aorto‐iliac aneurysmal disease (D'Oria & Mastrorilli, 2019; D'Oria & Mendes, 2020; D'Oria, Tenorio, Oderich, Mendes, et al., 2020; D'Oria & Tenorio, 2022; D'Oria & Tenorio, 2020; D'Oria & Lima, 2022; D'Oria & Pipitone, 2018; Lima et al., 2022; Schneider et al., 2023; Zuccon et al., 2023). Previous studies comparing the endovascular approach to IBE and traditional open surgery showed IBE deployment is associated with lower rates of major complications, shorter operative time and in‐hospital stay (D'Oria & Mendes, 2020). Its use is also safe and effective for the treatment of aneurysms in the IIA with selective cannulation of the posterior or anterior division of the IIA as distal landing zones (D'Oria, Tenorio, Oderich, Mendes, et al., 2020). Also, IBE can be used for the treatment of bilateral aneurysmal disease of the CIA with a technical success rate of 97% (provided appropriate anatomical requirements are met) (D'Oria & Tenorio, 2022). Where necessary, the posterior divisional branch of the IIA should be preferentially preserved as this maintains in‐line flow to the iliolumbar, lateral sacral and superior gluteal arteries. However, in cases of larger IIA aneurysms, there are risks to leaving patent side branches intact as they may lead to aneurysm sac enlargement through type 2 endoleaks.

Hence, it is important to recognize these vessels and perform appropriate embolization in order to prevent retrograde flow after implantation of the device. Additionally, the Gore Excluder IBE could be used as a standalone stent‐graft (although this application remains outside the manufacturer's IFU) for the treatment of isolated iliac artery aneurysms without the implantation of an aortic device, provided an appropriate sealing zone is present in the most proximal part of the CIA) (D'Oria & Tenorio, 2020; D'Oria & Lima, 2022; D'Oria & Pipitone, 2018). Zuccon et al. (2023) showed in their study that type Ib endoleaks correlated with an increased CIA diameter of >18 mm, length of distal landing zone <20 mm as well as increased iliac tortuosity. For the treatment of these type Ib endoleaks the use of IBE endoprothesis was suggested. These procedures proved to be safe and effective solutions with 95% freedom from IIA instability and 90% side branch patency in 60 months of follow‐up (Mastrorilli et al., 2023; Spath et al., 2023; Tenorio et al., 2019). In this case, cannulation of the device gate can be done with a multitude of technical approaches, including an antegrade upper arm approach or using a steerable sheath from the ipsilateral side. IBE was also used in urgent situations for the treatment of type Ib endoleaks with 100% technical success and without any procedural‐related complications such as IIA occlusion, stent fracture, thrombosis, stent migration or pelvic ischaemia (D'Oria & Chiarandini, 2018).

In addition, some have recommended use of the Gore IBE in tortuous iliac arteries because the continuous metallic stent support was deemed to be more resistant to kinking in cases of large IIA and the availability of hypogastric artery graft diameters of up to 14.5 mm. DeRoo et al. (2021) showed in a multicentre study that changes in the initial iliac anatomy after EVAR were associated with increased rates of adverse iliac events. The IBE is more conformable without changing the length and tortuosity of the iliac axis than its available counterparts reducing the chances of distal endoleak or limb thrombosis (Della Schiava et al., 2016). The meta‐analysis by Giosdekos et al. (2020) showed that the Gore IBE presented with an endoleak rate of 22%, which was slightly higher than with the other commercial IBDs studied. However, there was no impact on other clinical outcomes such as aneurysm enlargement or reintervention rates. It was hypothesized this may be due to improved imaging modalities (also considering that the Gore IBE was introduced in the market later as compared with the Cook Zenith IBD) and stricter selection of patients in earlier studies. However, these findings may also hide a potential selection bias that may not be entirely ruled out, since the choice of the IBE over alternative branch platforms could be related to certain anatomical characteristics like severe tortuosity of iliac vessels or short CIA (which are considered contraindications for the Cook Zenith and Jotec/Artivion Inc. E‐liac platforms). An excessive tortuosity as well as landing with the proximal part of IBE in the aneurysmal distal aorta (which is sometimes needed in case of short CIA) can represent a predisposing factor for component disconnection and secondary endoleaks with subsequent need for reinterventions.

## 4 FROM VASCULAR PHYSIOLOGY TO TECHNICAL TIPS: BRIDGING THE GAP FROM BENCH TO BEDSIDE

The Gore IBE is a versatile device providing a safe and effective solution for the treatment of AAA with extension of aneurysmal disease into the CIA. In pivotal trials, the primary patency rates of the internal iliac and external iliac branches were as high as 95% and 100%, respectively (which is another strength of the device especially in cases with severe iliac tortuosity where high conformability is indeed desired). The relatively low‐profile 16Fr sheath that is required for deployment makes a percutaneous approach feasible under most circumstances, with good trackability also in patients with narrower access vessels.

To ensure durable technical success several points must be considered, and extensive preoperative assessment of the anatomy, detailed knowledge of the device and careful intraoperative manipulation are all mandatory. Some useful tips to ensure safe deployment and sustained success, also in challenging anatomical situations, are listed and discussed below:

- Intraoperative measurement of the length between the IBE bifurcation and the aortic stent‐graft is necessary for precise deployment of the device. Also, optimal selection of bridging stents to achieve maximal overlapping without compromising the perfusion of the side branch. There is evidence of better conformability in the tortuous IIA with self‐expanding stents and distal relining using bare metal stents to ensure a smooth transition at angulation or over curves (Donas et al., 2012; Verzini et al., 2020). However, the recent development of the balloon expandable Gore Viabahn endoprosthesis (VBX) has shown excellent outcomes, and further research is needed (Cortolillo et al., 2023; Lima et al., 2022; Pickney et al., 2022). In effect, one of the main differences between the IBE device and other branch platforms is the diameter of the docking zone for the hypogastric stent, since the device is assigned to be used in conjunction with the dedicated hypogastric component. Also, when an upper extremity access is required, this may limit the use of the hypogastric component because of the limited length of the shaft. With this in mind, the use of different covered stents should be carefully weighed because of the potential mismatching between the portal diameter and the distal landing zone, especially in case of a concomitant IIA aneurysm requiring landing into divisional branches. Nonetheless, a recent European multicentric study (HYPROTECT) (D'Oria et al., 2024) has shown that use of the IBE in conjunction with different combinations of stent‐grafts from the same manufacturer has primary stability of the iliac branch as high as 90% in the setting of hypogastric aneurysms, although caution seems necessary when multiple bridging stent‐grafts are used as this may predispose to increased rates of instability and reinterventions.
- Cannulation of the IIA can be challenging in the presence of iliac artery tortuosity, severe ostium angulation of the IIA, iliac stenosis and elongated aneurysms. Unobstructed run‐off of the side branch is necessary for the longevity and patency of any reconstruction. Kinking of the side branch, prior occlusion of the IIA, poor run‐off, and selection of long but narrow side branch stents should be avoided or, if present, adhere to a stricter follow‐up.
- Kissing balloon angioplasty at the iliac bifurcation after deployment of both components of the IBE should be performed. This ensures adequate moulding of the stent‐grafts and minimizing possible compromise of the side branch from the main device. If the distal landing zone in the IIA is inadequate, the side branch can be deployed into healthy segments of the main division branches. This may constitute a direct violation of IFU but remains a compromise in the treatment of complex cases with aneurysmal internal iliac arteries.
- Meticulous preoperative sizing and planning of the procedure constitute the basis for a successful EVAR procedure with adjunctive IBE. The multimodular device, although it has quite strict IFU, has advantages in those with complex anatomy. A recent study showed that 60% of procedures with IBE are performed outside of IFU without adverse events (Schneider et al., 2018). It is essential to plan treatment in order to allow for a reconstruction that can be as physiological as possible. This entails ensuring an optimal match between all implanted devices and the underlying vessels, assessing the anatomical characteristics (e.g., size and tortuosity) as well as their physiological constraints (e.g., mobility). Further integration between advanced computer‐aided simulation of blood flow modification after endovascular treatment and detailed assessment of long‐term results may enable critical assessment of modes of failure that will help to ameliorate the quality of care delivered.
- Long‐term outcomes may also be impacted by the type and duration of antithrombotic therapy that is prescribed to patients. While more intensive regimes may lead to improved patency rates, they could also expose patients at higher risks of bleeding, and a careful balance must therefore be sought. There is a lack of detailed level 1 evidence concerning this but a similar approach should be adopted as to what is done following stenting of reno‐mesenteric vessels (D'Oria & Bertoglio, 2022). Unless the patient is already on chronic oral anticoagulation for concomitant medical reasons, a 3‐ to 6‐month course of dual antiplatelet therapy (DAPT) could be recommended, followed by lifelong single antiplatelet therapy. However, when a distal landing zone is in smaller divisional branches of the IIA then an extended course of DAPT or a switch in antithrombotic therapy might be considered. Also, further research is required concerning some unresolved issues, such as the identification of patients who are non‐responders to antiplatelet drugs (Kankaria et al., 2024).

## 5 CONCLUSIONS

Endovascular aneurysm repair with adjunctive IBE implantation is a safe and effective solution for the treatment of complex AAA with adjunctive aneurysmal disease of the CIA and inadequate distal landing zone. The current literature shows evidence of extremely high technical success rates and similar long‐term results to traditional open surgery. However, preservation of the pelvic circulation, as opposed to less physiological interruption of the pelvic blood flow via embolization of the IIA (and its branches), is recommended to reduce symptoms of pelvic ischaemia. This can also be performed bilaterally provided certain anatomical requirements are met.

## AUTHOR CONTRIBUTIONS

Conception and design: Apostolos G. Pitoulias, Mario D΄Oria, Konstantinos P. Donas, MB. Analysis and interpretation: Apostolos G. Pitoulias, Mario D΄Oria, Konstantinos P. Donas, Matti Jubouri, Damian M. Bailey, Ian M. Williams, and Mohamad Bashir. Data collection: Apostolos G. Pitoulias and Mario D΄Oria. Writing the manuscript: Apostolos G. Pitoulias and Mario D΄Oria. Critical revision: Apostolos G. Pitoulias, Mario D΄Oria, Konstantinos P. Donas, Matti Jubouri, Damian M. Bailey, Ian M. Williams, and Mohamad Bashir. All authors have read and approved the final version of this manuscript and agree to be accountable for all aspects of the work in ensuring that questions related to the accuracy or integrity of any part of the work are appropriately investigated and resolved. All persons designated as authors qualify for authorship, and all those who qualify for authorship are listed.

## CONFLICT OF INTEREST

The authors declare no conflict of interest.
